# Transcranial Doppler ultrasonography predicts cardiovascular events after TIA

**DOI:** 10.1186/1471-2342-9-13

**Published:** 2009-07-30

**Authors:** Katrin Holzer, Suwad Sadikovic, Lorena Esposito, Angelina Bockelbrink, Dirk Sander, Bernhard Hemmer, Holger Poppert

**Affiliations:** 1 Department of Neurology, Klinikum rechts der Isar, Technische Universität, Munich, Germany; 2 Institute for Social Medicine, Epidemiology, and Health Economics, Charité University Medical Centre, Berlin, Germany; 3 Department of Neurology, Medical Park Hospital, Bischofswiesen, Germany

## Abstract

**Background:**

Transient ischemic attack (TIA) patients are at high vascular risk. We assessed the value of extracranial (ECD) and transcranial (TCD) Doppler and duplex ultrasonography to predict clinical outcome after TIA.

**Methods:**

176 consecutive TIA patients admitted to the Stroke Unit were recruited in the study. All patients received diffusion-weighted imaging, standardized ECD and TCD. At a median follow-up of 27 months, new vascular events were recorded.

**Results:**

22 (13.8%) patients experienced an ischemic stroke or TIA, 5 (3.1%) a myocardial infarction or acute coronary syndrome, and 5 (3.1%) underwent arterial revascularization. ECD revealed extracranial ≥ 50% stenosis or occlusions in 34 (19.3%) patients, TCD showed intracranial stenosis in 15 (9.2%) and collateral flow patterns due to extracranial stenosis in 5 (3.1%) cases. Multivariate analysis identified these abnormal ECD and TCD findings as predictors of new cerebral ischemic events (ECD: hazard ratio (HR) 4.30, 95% confidence interval (CI) 1.75 to 10.57, P = 0.01; TCD: HR 4.73, 95% CI 1.86 to 12.04, P = 0.01). Abnormal TCD findings were also predictive of cardiovascular ischemic events (HR 18.51, 95% CI 3.49 to 98.24, P = 0.001).

**Conclusion:**

TIA patients with abnormal TCD findings are at high risk to develop further cerebral and cardiovascular ischemic events.

## Background

After a transient ischemic attack (TIA), patients are at high risk to develop further vascular events. The risk of stroke within the first 90 days after TIA is 4% to 20%, with half of the events occurring within the first 2 days [1-6]. Consequently, the early risk of stroke after TIA is comparable to, or even higher than, the short-term risk of myocardial infarction (MI) and major cardiovascular complications in patients presenting with chest pain [7]. Several clinical characteristics such as advanced age [2,8-10], diabetes mellitus [2,9], hypertension [9,10], weakness [2,9,10], speech impairment [2,9,10], prolonged symptom duration [2,8-10], evidence of acute ischemia on brain imaging [3,6,11,12], extracranial or intracranial large-artery occlusive disease [3,6,13-15], and cardioembolism [14], have been reported to be independently associated with a higher incidence of early subsequent stroke after TIA. Recently a new scoring system for evaluating short-term stroke risk after TIA (ABCD^2 ^score) based on 5 clinical factors has been validated [9,16,17]. The predictive power of the ABCD^2 ^model seems to be partially explained by identification of those patients likely to have experienced a true TIA [18].

Whereas the risk of stroke is highest in the first year after TIA, ranging from 7% to 21% [1,5,8,19,20], and afterwards declines to an annual rate of 2% to 6% over the first 4 to 5 years [19-21], the annual risk of coronary events after TIA remains stable at about 2% to 3% for several years [19,20]. On long-term follow-up, cardiovascular disease becomes the major cause of death after TIA [22]. The 10-year risk of vascular events in TIA patients is reported to be 36% and the 10-year risk of death 34% respectively [23]. Several studies have already demonstrated a high prevalence of asymptomatic coronary artery disease (CAD) in patients with TIA and mild ischemic stroke (IS), ranging from 28% to 41% [24-26]. Routine screening tests for CAD in all patients with cerebrovascular disease may not be cost-effective, however. Healthcare professionals currently are encouraged to optimize coronary risk evaluation in patients with TIA and IS based on the individual cardiovascular risk profile and the prevalence of carotid artery disease [27].

In this study, we aimed to assess the value of extracranial (ECD) and transcranial (TCD) Doppler and duplex ultrasonography to predict the occurrence of cerebrovascular and cardiovascular events after TIA.

## Methods

We identified 262 patients with possible cerebral TIA, who had been consecutively admitted to the Stroke Unit of the Department of Neurology, Technical University Munich, within the first 72 hours after symptom onset between May 2000 and July 2004. Diagnosis was made by the attending neurologist before patient selection. TIA was defined as an acute transient focal neurological deficit caused by vascular disease, which completely reversed within 24 hours [28]. Patients with amaurosis fugax were not included in the study, as data suggest pathogenic and prognostic differences between transient eye and brain ischemic syndromes [29,30].

To be eligible, patients had to undergo cerebral magnetic resonance imaging (MRI) including diffusion-weighted imaging (DWI) sequences within 5 days after onset of symptoms, which was the case in 225 patients. 49 patients were excluded for the following reasons: competing differential diagnosis as assessed by the attending neurologist, 41 cases (migraine, 8 cases; epilepsy, 7 cases; functional disorder, 5 cases; peripheral dizziness, 4 cases; syncope, 4 cases; hypertensive crisis, 4 patients; others, 9 cases); malignancy requiring active treatment, 7 cases; concomitant participation in a pharmaceutical trial; 1 case. Informed consent was obtained of every patient.

Routine admission examinations involved evaluation of medical history, physical examinations, blood analysis including lipid and glucose metabolism, resting and 24-hour electrocardiogram, 24-hour blood pressure measurement, transthoracic echocardiography, ECD, TCD, and cerebral MRI including DWI sequences. Symptom duration was systematically documented.

The following baseline clinical data were collected: age, sex, symptom duration, presence of classic vascular risk factors, and medical history of CAD, cardiac failure, and peripheral artery disease (PAD) (Additional file 1). Hypertension was defined as systolic blood pressure ≥ 140 mmHg, diastolic blood pressure ≥ 90 mmHg, or current use of antihypertensive medication; diabetes mellitus as fasting blood glucose ≥ 126 mg/dL or current use of antidiabetic agents; and hypercholesterolemia as total cholesterol ≥ 240 mg/dL or current use of lipid-lowering medication. Nicotine abuse was defined as current or former regular smoking. Atrial fibrillation was defined as history of electrocardiographically documented intermittent or persistent atrial fibrillation. TIA was allocated to the carotid or vertebrobasilar territory by an experienced neurologist based on clinical symptoms and MRI.

ECD and TCD were performed within a maximum of 3 days after admission using multi-range doppler (DWL Multi-Dop; Compumedics Germany GmbH) and duplex ultrasound devices (Siemens Sonoline Elegra; Siemens AG).

ECD findings were classified as follows: normal (1), if there was no evidence of plaques both in the cervical internal carotid (cICA) and cervical vertebral arteries (cVA); atherosclerosis without stenosis (2), if cICA or cVA showed at least one plaque with < 50% stenosis of the corresponding vessel; stenosis (3), if cICA or cVA showed at least one ≥ 50% stenosis or an occlusion. ECD classification did not distinguish between symptomatic and asymptomatic vessel disease.

TCD findings were classified as follows: normal (1), if TCD detected no pathological findings or only minor side-to-side differences of the distal internal carotid (dICA), middle cerebral (MCA), posterior cerebral (PCA), or intracranial vertebrobasilar (VBA) artery; reactive collateral flow patterns (2), if TCD demonstrated collateral blood flow through the circle of Willis secondary to extracranial lesions; stenosis (3), if dICA, MCA, PCA, or VBA showed at least one intracranial stenosis or an occlusion. TCD diagnosis of intracranial stenosis was defined by increased peak flow velocities (≥ 155 cm/s for dICA and MCA; ≥ 100 cm/s for PCA and VBA) with side-to-side differences > 20% and disturbed flow patterns [31,32]. PCA signal was identified by posterior angulation of the probe during transtemporal insonation using an insonation depth of 60 to 70 mm. For identification of VBA we applied an insonation depth of 60-79 mm (intracranial vertebral arteries) and 80-110 mm (basilar artery) during suboccipital insonation. TCD classification did not distinguish between symptomatic and asymptomatic vessel disease.

Cerebral MRI was performed within a maximum of 5 days after symptom onset in all patients. No patient developed a follow-up cerebrovascular event before MRI.

All MRI scans were obtained using a 1.5-Tesla scanner (Magnetom Symphony; Siemens AG). Imaging protocol included axial T1-weighted (TR/TE 654/14 ms), T2-weighted (TR/TE 3305/132 ms), and DWI sequences (TR/TE 4006/83 ms, slice thickness 4 to 6 mm, interslice gap 1.5 mm, pixel matrix 128 × 128, field of view 220 × 220 mm, pixel size 1.72 × 1.72 mm, gradient strength 30 mT/m, b-values = 0, 500, 1000 s/mm^2^), and in doubtful cases additionally a sagittal or coronal DWI sequence. ADC maps were constructed by linear least-squares fit on a pixel-by-pixel basis after averaging the direction-dependent DWI values.

DWI scans were considered positive for ischemia if both a hyperintensity on the isotropic b = 1000 scan and a corresponding hypointensity on the ADC map were detectable.

At a median follow-up of 27 months (minimum 4 months, maximum 64 months) all 176 patients were contacted via telephone or mail by an experienced neurologist blinded to the patients' ultrasonographic findings. A semi-structered interview was used to assess new cerebral ischemic or other vascular events. If the interview provided insufficient data or any indication of follow-up events, data were completed by contacting relatives, attending physicians and/or hospitals. Our main points of interest were cerebral ischemic events (ischemic stroke or TIA), cardiovascular ischemic events (MI or acute coronary syndrome (ACS), surgical or endovascular revascularization procedures in CAD or PAD), and death of vascular or unknown cause. Other vascular events and death of nonvascular cause also were documented. If a patient reported symptoms possibly consistent with a follow-up event but did not seek medical aid or had competing differential diagnoses as reported by the attending physician, this information was documented but not considered as an outcome event.

All analyses were performed with the SPSS statistical package version 15.0. Univariate Cox regression analysis was used to detect variables associated with the occurrence of endpoints. Cox proportional hazards multivariate analysis adjusted for age and sex was applied to identify independent predictors of cerebral ischemic events, cardiovascular ischemic events, and the combined endpoint of cerebral ischemic events, cardiac ischemic events, and death of vascular or unknown cause. *P *< 0.05 was considered as significant. Percentage values are relative to the patent subset with complete data record.

The research protocol was approved by the local ethical committee.

## Results

A total of 176 Caucasian TIA patients were included in the study. Table 1 shows the baseline characteristics of the study population. Medical history revealed former IS, TIA, or amaurosis fugax in 40 (23.1%) patients. 9 (5.1%) patients reported a TIA during the last month before admission.

**Table 1:** Baseline characteristics of study population (n = 176)

Age (y)*	63.3 ± 14,5
Sex, female (n)	67 (38.1%)
Hypertension (n)	127 (72.2%)
Diabetes mellitus (n)	28 (15.9%)
Hypercholesterolemia (n)	84 (48.6%)
Body mass index*	25.8 ± 3.9
Nicotine abuse (n)	80 (45.5%)
Atrial fibrillation (n)	24 (13.6%)
Coronary artery disease (n)	35 (19.9%)
Cardiac failure (n)	11 (6.4%)
Peripheral artery disease (n)	13 (7.4%)
DWI abnormality (n)	49 (28.3%)
Duration (h)*	4.7 ± 7.1
Duration ≥ 1 h and/or DWI abnormality (n)	123 (72.4%)
Vertebrobasilar TIA (n)	42 (23.9%)
ECD: stenoocclusion (n)	34 (19.3%)
TCD: abnormal (n)	20 (12.3%)

*Mean ± standard deviation.

Mean symptom duration was 4.7 ± 7.1 hours, with 65 (37.6%) patients having symptoms lasting < 1 hour. TIA was allocated to the carotid territory in 125 (71.0%) patients and to the vertebrobasilar territory in 42 (23.9%) patients; 9 further cases (5.1%) could not be classified on the basis of symptoms and MRI. DWI showed signal intensity changes suggestive of cerebral ischemia in 49 (28.3%) patients.

ECD detected plaques without stenosis in 84 (47.7%) patients and ≥ 50% stenosis or occlusions in 34 (19.3%) patients. 24 (13.6%) patients showed a stenosis of the cICA, 3 (1.7%) an occlusion of the cICA, 4 (2.3%) a stenosis of the cVA, 2 (1.1%) an occlusion of the cVA, and 1 (0.6%) stenosis or occlusions of both the cICA and cVA. 6 (3.4%) patients had a high-grade cICA stenosis of ≥ 80%. 5 of these patients underwent subsequent carotid endarterectomy and 1 patient stent-supported angioplasty.

TCD detected intracranial stenosis in 15 (9.2%) patients and reactive collateral blood flow due to stenosis of the cICA in 5 (3.1%) patients. 9 (5.1%) patients showed a stenosis of the dICA or MCA, 1 (0.6%) an occlusion of the dICA or MCA, 3 (1.7%) a stenosis of the PCA, 1 (0.6%) a stenosis of the VBA, and 1 (0.6%) stenosis of both the cICA or MCA and PCA. In 13 (7.4%) patients, TCD could not be applied because of inadequate temporal bone windows.

Tandem lesions of both the cICA and ipsilateral dICA or MCA were detectable in 3 (1.8%) patients.

### Follow-up endpoints

Follow-up data was available of 173 (98.3%) patients. 9 (5.7%) patients suffered an IS and 14 (8.8%) a further TIA; 9 (5.7%) more patients reported symptoms possibly consistent with cerebral ischemia but did not seek medical aid or had competing differential diagnoses as reported by the attending physician. In 7 of 14 patients with follow-up TIA, a new MRI was performed, which detected an acute ischemic lesion in only 1 patient.

3 (1.8%) patients experienced a MI and 2 (1.2%) an ACS during follow-up; a further 4 (2.4%) patients underwent surgical or endovascular revascularization in CAD, and 1 (0.6%) patient had bypass surgery in PAD. Additionally, 4 (2.4%) patients suffered from their first-ever angina pectoris attack, and 10 (6.0%) patients experienced other vascular events (cardiac syncope, 4 cases; pacemaker implantation, 2 cases; aortic valve surgery, 1 case; Wolff-Parkinson-White syndrome, 1 case; deep vein thrombosis, 1 case; pulmonary embolism, 1 case). 15 (8.5%) patients died due to the following reasons: cardiac failure, 3 (1.7%) cases; malignancy, 3 (1.7%) cases; pneumonia, 2 (1.1%) cases; unknown cause, 7 (4.0%) cases.

**Figure 1:**
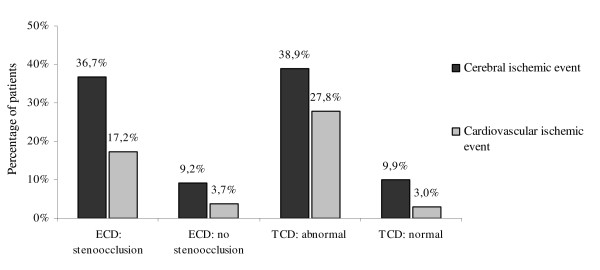
**Graded risk of new vascular events based on ECD and TCD findings**.

Figure 1 shows the graded risk of new cerebral ischemic events and cardiovascular ischemic events based on ECD and TCD findings.

### Predictors of new cerebral ischemic events

In univariate analysis (Table 2), detection of stenosis by ECD (hazard ratio (HR) 4.39, 95% CI 1.93 to 9.99, *P *< 0.01), evidence of reactive collateral flow patterns or intracranial stenosis by TCD (HR 4.99, 95% CI 1.97 to 12.62, *P *< 0.01), and PAD (HR 7.64, 95% CI 2.96 to 19.71, *P *< 0.01) were significantly associated with follow-up IS or TIA. A trend that did not reach significance was also found for the parameters prolonged symptom duration (HR 1.04, 95% CI 1.00 to 1.10, *P *= 0.07), advanced age (HR 1.03, 95% CI 0.99 to 1.06, *P *= 0.11), and cardiac failure (HR 3.39, 95% CI 1.00 to 11.55, *P *= 0.05). Evidence of acute ischemia on DWI had no significant effect in our study. Cox proportional hazards multivariate analysis (Table 3) confirmed pathological ECD and TCD findings (ECD: HR 4.30, 95% CI 1.75 to 10.57, *P *= 0.01; TCD: HR 4.73, 95% CI 1.86 to 12.04, *P *= 0.01) to be predictors of new cerebral ischemic events.

**Table 2:** Univariate analysis of variables possibly associated with new vascular events

	Cerebral ischemic event		Cardiovascular ischemic event		Cerebral/cardiovascular ischemic event or vascular/unknown death	
	
	HR	95% CI	HR	95% CI	HR	95% CI
Age	1.03	0.99-1.06	1.04	0.99-1.10	1.04	1.01-1.07
Sex, female	1.13	0.49-2.62	0.93	0.23-3.76	1.18	0.56-2.45
Hypertension	0.99	0.39-2.51	-*	-*	1.20	0.54-2.68
Diabetes	2.11	0.83-5.35	5.00	1.40-17.86	1.85	0.82-4.17
Hyperlipidemia	0.57	0.24-1.34	2.51	0.65-9.70	0.51	0.24-1.10
Body mass index > 30	0.30	0.04-2.24	1.06	0.21-5.26	0.52	0.15-1.76
Nicotine abuse	1.30	0.57-2.94	0.99	0.27-3.69	1.06	0.53-2.12
Atrial fibrillation	0.94	0.28-3.17	-*	-*	1.02	0.35-2.93
Coronary artery disease	0.59	0.18-1.99	2.27	0.62-8.24	0.96	0.41-2.24
Cardiac failure	3.39	1.00-11.55	2.77	0.34-22.63	3.97	1.51-10.45
Peripheral artery disease	7.64	2.96-19.71	-*	-*	7.42	3.25-16.94
DWI abnormality	1.23	0.51-2.93	0.59	0.12-2.85	0.84	0.37-1.90
Duration	1.04	1.00-1.10	0.92	0.79-1.08	1.03	0.99-1.08
Duration ≥ 1 h and/or DWI abnormality	0.76	0.31-1.89	0.69	0.17-2.76	0.91	0.40-2.07
Vertebrobasilar TIA	0.17	0.02-1.24	1.33	0.32-5.46	0.39	0.12-1.30
ECD: stenoocclusion	4.39	1.93-9.99	3.73	1.05-13.31	4.18	2.04-8.59
TCD: abnormal	4.99	1.97-12.62	9.62	2.46-37.68	5.13	2.26-11.67

* Statistical analysis not possible due to small patient numbers.

SD: Standard deviation. HR: Hazard ratio. CI: Confidence interval.

**Table 3:** Predictors of new vascular events in multivariate analysis*

	Cerebral ischemic event		Cardiovascular ischemic event		Cerebral/cardiovascular ischemic event or vascular/unknown death	
	
	HR	95% CI	HR	95% CI	HR	95% CI
ECD: stenoocclusion	4.30	1.75-10.57	2.93	0.77-11.17	3.46	1.56-7.66
TCD: abnormal	4.73	1.86-12.04	18.51	3.49-98.24	4.97	2.16-11.47

* Cox proportional hazards multivariate analysis adjusted for age and sex.

HR: Hazard ratio. CI: Confidence interval.

### Predictors of new cardiovascular ischemic events

Detection of extracranial stenosis by ECD (HR 3.73, 95% CI 1.05 to 13.31, *P *= 0.04), proof of reactive collateral flow patterns or intracranial stenosis by TCD (HR 9.62, 95% CI 2.46 to 37.68, *P *< 0.01), and diabetes mellitus (HR 5.00, 95% CI 1.40 to 17.86, *P *= 0.01) were significantly associated with the occurrence of MI, ACS, or revascularization procedures in univariate analysis (Table 2). However, whereas abnormal TCD findings (HR 18.51, 95% CI 3.49 to 98.24, *P *= 0.001) proved to predict cardiovascular ischemic events in multivariate analysis (Table 3), pathological ECD findings (HR 2.93, 95% CI 0.77 to 11.17, *P *= 0.116) failed to reach significance in multivariate analysis.

### Predictors of the combined endpoint of cerebral ischemic events, cardiac ischemic events, and death of vascular or unknown cause

In univariate analysis, detection of extracranial stenosis by ECD (HR 4.18, 95% CI 2.04 to 8.59, *P *< 0.01), evidence of reactive collateral flow patterns or intracranial stenosis by TCD (HR 5.13, 95% CI 2.26 to 11.67, *P *< 0.01), advanced age (HR 1.04, 95% CI 1.01 to 1.07, *P *< 0.01), PAD (HR 7.42, 95% CI 3.25 to 16.94, *P *< 0.01), and cardiac failure (HR 3.97, 95% CI 1.51 to 10.45, *P *< 0.01) were significantly associated with the combined endpoint of IS or TIA, MI or ACS, and death of vascular or unknown cause. Both pathological ECD and TCD findings (ECD: HR 3.46, 95% CI 1.56 to 7.66, *P *= 0.02; TCD: HR 4.97, 95% CI 2.16 to 11.47, *P *< 0.001) proved to be predictors of the combined endpoint in multivariate analysis (Table 3).

## Discussion

The present study demonstrates that TIA patients with ultrasonographic evidence of extracranial or intracranial stenoocclusive disease are at high risk of further cerebral ischemic events during medium- to long-term follow-up. After a median follow-up of 27 months, nearly 40% of the patients with either stenoocclusive disease in ECD or pathological findings in TCD have suffered a new IS or TIA. Several studies have already reported an increased stroke incidence after TIA of large-artery atherosclerotic or cardioembolic etiology as compared to other subtypes, but to our knowledge only for short- and medium-term follow-up [3,13-15]. Purroy et al., in this context, have shown a 3-month stroke risk of 20% after TIA due to large-artery atherosclerosis [14]. The present data demonstrate that detection of stenoocclusive disease by ECD or TCD is not only associated with a higher short-term stroke risk after TIA but remains a predictor of recurrent cerebral ischemia during medium- to long-term follow-up.

Whereas several previous studies found a significant association between prolonged symptom duration and recurrent cerebral ischemic events during short- and medium-term follow-up after TIA [2,8-10,13], there was only a trend that did not reach significance in this study. We also could not show that TIA patients with acute ischemia on DWI are at higher risk of further cerebral ischemic events on medium- to long-term follow-up. Because of the limited number of patients, however, these findings may be simply explained by chance.

As an additional result of the present study, vertebrobasilar TIA allocation seems to be associated with a lower recurrence rate of cerebral ischemia. This finding is consistent with a recent systematic review of Flossmann et al., who noted a lower stroke incidence in patients with vertebrobasilar TIA or minor stroke when data was confined to hospital-based studies (OR 0.68, 95% CI 0.6 to 0.8), but a higher stroke incidence when data was restricted to population-based studies (OR 1.48,95% CI 1.1 to 2.0) [33].

A second major finding of the present study is that detection of reactive collateral flow patterns or intracranial stenosis by TCD predicts new cardiovascular ischemic events on medium- to long-term follow-up after TIA. 5 of 18 (27.8%) patients with abnormal TCD findings, but only 4 of 134 (3%) patients without, have developed a subsequent cardiovascular ischemic event. The association between TCD findings and cardiovascular prognosis is of particular importance as cardiovascular disease becomes the major cause of death on long-term follow-up after TIA [22].

A high prevalence of asymptomatic CAD in patients with cerebrovascular disease is well known [27]. Chimowitz et al. documented abnormal cardiac stress tests in 50% of TIA or stroke patients with large-artery occlusive disease [34], with the rates being 25% in isolated intracranial artery stenosis, 50% in isolated extracranial carotid stenosis, and even 83% in coexistent extracranial carotid and intracranial artery stenosis [34]. In another study, symptomatic intracranial atherosclerosis was associated with a 52% risk of occult CAD [35]. However, whereas a strong correlation between the extent of extracranial carotid and coronary atherosclerosis is well accepted in Caucasians [36], and guidelines already recommend coronary risk evaluation in TIA patients based on the individual cardiovascular risk profile and the prevalence of carotid artery disease [27], the relationship between intracranial atherosclerosis and CAD has not been sufficiently evaluated in this ethnic group. In Asians, who suffer from intracranial atherosclerosis more frequently than Caucasians, correlation between extracranial carotid and coronary atherosclerosis seems to be stronger than between intracranial artery and coronary atherosclerosis [37].

Whereas pathological TCD findings proved to be a predictor of new cardiovascular ischemic events in the present study, detection of stenoocclusive disease by ECD failed significance in multivariate analysis. However, as our definition of stenoocclusive disease in ECD included both cICA and cVA lesions, the results cannot be equated with the prognostic value of isolated extracranial carotid disease, which might be higher. Moreover, the definition of pathological TCD findings referred to both reactive collateral blood flow secondary to extracranial lesions and intracranial stenoocclusive disease in this study, thus probably characterizing those TIA patients with the highest risk of generalized atherosclerosis and consequently cardiovascular ischemic events. TCD is already accepted as an accurate, safe, and cost-effective diagnostic tool for the detection of intracranial stenoocclusive disease[38,39], and is widely accessible in most countries. The results of the present study support the routine use of TCD in addition to ECD in TIA patients. Moreover, routine screening tests for CAD and aggressive prevention therapies should be considered in TIA patients with pathological TCD findings.

The present study has several limitations. A larger patient cohort would have been necessary to improve the statistical power of the study and allow further subgroup analyses. Moreover, follow-up was conducted as telephone or mail interview only. Even though attending physicians and/or hospitals were contacted to complete data, early and minor vascular events may have been missed due to limited ability of the patients to recall their symptoms over the complete follow-up period. A further weakness of the present study is the lack of angiographic validation of ECD and TCD findings. However, both ECD and TCD have been shown to be accurate diagnostic tools for the detection of extracranial and intracranial stenoocclusive disease [38-40]. Finally, ECD and TCD classification did not distinguish between symptomatic and asymptomatic vessel disease in the present study.

## Conclusion

The present study confirms the high risk of further cerebral ischemic events in TIA patients with ultrasonographic evidence of extracranial or intracranial stenoocclusive disease during medium- to long-term follow-up. As pathological TCD findings additionally predict new cardiovascular ischemic events, routine screening tests for CAD and aggressive prevention therapies should be considered in this subgroup of TIA patients. ECD and TCD are important diagnostic procedures in patients with TIA.

## Competing interests

The authors declare that they have no competing interests.

## Authors' contributions

KH carried out the data collection and drafted the manuscript. SS participated in its design and data collection. LE participated in the follow-up data collection and has been involved in drafting the manuscript. AB performed the statistical analyses. DS revised the manuscript critically for important intellectual content and helped to draft the manuscript. BH made substantial contributions to conception, revised the manuscript and gave final approval of the version to be published. HP conceived of the study, and participated in its design and coordination and helped to draft the manuscript. All authors read and approved the final manuscript.

## Pre-publication history

The pre-publication history for this paper can be accessed here:

http://www.biomedcentral.com/1471-2342/9/13/prepub

## Supplementary Material

Additional file 1 **Baseline characteristics of patients with and without abnormal TCD**. The data provided represent the baseline characteristics of the patients with and without abnormal TCD. Click here for file
