# Supplementary material for: Transcranial Doppler ultrasonography predicts cardiovascular events after TIA
Source: BMC Med Imaging. 2009 Jul 30;9:13. doi: 10.1186/1471-2342-9-13 (PMC2730052; doi:10.1186/1471-2342-9-13)
Supplement: Additional file 1 — Baseline characteristics of patients with and without abnormal TCD. The data provided represent the baseline characteristics of the patients with and without abnormal TCD. [file 1471-2342-9-13-S1.doc]

**Additional table. Baseline characteristics of patients with and without abnormal TCD**

|  | TCD: abnormal (n = 20) | TCD: normal  (n = 143) | *P* |
| --- | --- | --- | --- |
| Age (y)* | 65.2 ± 12.5 | 62.1 ± 14.7 | 0.37 |
| Sex, female (n) | 8 (40.0%) | 50 (35.0%) | 0.66 |
| **Hypertension (n)** | **18 (90.0%)** | **98 (68.5%)** | **0.047** |
| Diabetes mellitus (n) | 6 (31.6%) | 19 (13.5%) | 0.08 |
| Hypercholesterolemia (n) | 14 (70.0%) | 66 (47.1%) | 0.06 |
| Body mass index* | 25.1 ± 2.6 | 26.1 ± 4.0 | 0.32 |
| Nicotine abuse (n) | 10 (50.0%) | 67 (46.9%) | 0.79 |
| Atrial fibrillation (n) | 1 (5.3%) | 19 (13.6%) | 0.47 |
| Coronary artery disease (n) | 7 (35.0%) | 25 (17.7%) | 0.08 |
| Cardiac failure (n) | 2 (10.0%) | 8 (5.7%) | 0.36 |
| Peripheral artery disease (n) | 3 (15.0%) | 7 (4.9%) | 0.11 |
| DWI abnormality (n) | 6 (30.0%) | 39 (27.3%) | 0.84 |
| Duration (h)* | 4.0 ± 6.4 | 4.8 ± 7.2 | 0.65 |
| Duration ≥1 h and/or  DWI abnormality (n) | 12 (60.0%) | 103 (74.1%) | 0.19 |
| Vertebrobasilar TIA (n) | 6 (31.6%) | 34 (24.5%) | 0.58 |

*Mean ± standard deviation.

Association of risk factors was assessed by Student t test for normally distributed data and χ^2^ test or Fisher`s exact test for categorized variables.
